# Electronic Gaming Machine Consumers’ Understanding of Past & Future Spending: Associations with Risk, Impulsivity, Self-Control, & Problematic Gambling

**DOI:** 10.1007/s10899-025-10405-y

**Published:** 2025-06-09

**Authors:** Teejay Santos, Robert M. Heirene, Deborah Cobb-Clark, Agnieszka Tymula, Sally M. Gainsbury

**Affiliations:** 1 Brain & Mind Centre, School of Psychology, Science Faculty, University of Sydney, Sydney, Australia; 2 ARC Centre of Excellence for Children and Families Over the Life Course, School of Economics, Faculty of Arts & Social Sciences, University of Sydney, Sydney, Australia; 3 Institute of Labor Economics (IZA), Bonn, Germany

**Keywords:** Gambling disorder, Risky behaviour, Impulsivity, Self-control, Decision-making, Problem gambling

## Abstract

An individual’s ability to make informed decisions about future gambling expenditure requires an awareness of affordability and likely return, and hence an accurate understanding of their past outcomes. Electronic gaming machines (EGMs) have been criticised for having structural characteristics that erode informed decision-making (e.g., loses disguised as wins, display of credits vs. currency). Here we investigate individual’s understanding of their recent and future  EGM spending. Survey responses for 187 Australian EGM customers (age range:18–81, 57% female) were linked to their account data held by a gambling venue. Few customers accurately recalled and predicted their past/future 30-day expenditure to within a 10% margin of error, including net outcome (i.e., accurate recall [5.5%] or prediction [17.4%] of total amount won or lost); win (10.5%; 32.9%), and total spend (2.6%; 12.8%). The ability to accurately report gambling expenditure was almost exclusively limited to those who did not gamble during the period reported. Participants with greater impulsivity and more willingness to take risks were less accurate in their prediction of net outcome and spend, respectively. Across recall and prediction, participants commonly overestimated their net result, yet underestimated the amount they won, suggesting that winnings are re-gambled without considering them as their own funds. Our findings demonstrate EGM customers do not understand their gambling expenditure and changes are needed to the product and environment to facilitate informed choice.

**Supplementary Information:**

The online version contains supplementary material available at 10.1007/s10899-025-10405-y.

## Introduction

Informed decisions about whether to gamble and how much to spend involve weighing up the potential risks and rewards for each bet placed. As with most consumer decisions, understanding the product and associated risks is considered the customer’s responsibility. However, gambling operators—like energy companies, loan providers, healthcare services, and other service providers—have a responsibility to provide their customers with the necessary information to facilitate informed decision-making (Blaszczynski et al., 2004; Hancock et al., 2008; Wohl et al., 2017). Current evidence links problematic gambling with impaired decision-making; at-risk individuals gravitate towards high-risk and high-reward choices (Linnet et al., 2010, 2011). Consequentially, most gambling regulators in Australia require licensed operators to provide customers with sufficient information to make an informed decision about their gambling. This can take the form of ‘return to player’ statements on machines outlining the overall, long-term expected return of the machine (e.g., *"This game has an average percentage payout of 90%"*), as well as warnings about the potential risks associated with gambling (e.g., *“Chances are you’re about to lose”*; Newall et al., 2022; Newall et al., 2023b).

Electronic gaming machines (EGMs; also referred to as pokies, slot machines, and video lottery terminals) are of central interest to gambling regulators and researchers, given their strong connection with gambling problems (Delfabbro et al., 2020). The structural characteristics of EGMs may erode informed decision-making and lead to excessive gambling. For example, the lack of a natural break in play encourages continuous gambling. Having bets across multiple ‘lines’ within a single spin making it possible to achieve and have the machine celebrate ‘wins’ even if these are less than the amount bet (i.e., “losses disguised as wins”) and the complexity of multi-line machines may make it appear as if a win was ‘nearly’ achieved thus propagating continued efforts. Awareness of spend can be distorted as wins are automatically credited rather than paid out, bets can be presented in credits rather than in currency value, and bets are funded by cash and/or tickets without any receipts to clarify total spend. Studies show EGMs and the rates at which they pay-out are systematically misunderstood, resulting in cognitive biases that relate strongly to more positive attitudes toward EGMs (Philander & Gainsbury, 2021). Positive attitudes have been shown to predict intent to play EGMs and individuals who are overconfident that they understand EGMs may be more likely to use them (Philander & Gainsbury, 2021). A poor understanding of EGMs and related cognitive biases are related to gambling problems (Gainsbury et al., 2020; Goodie, 2005; Philander & Gainsbury, 2023). Commonly held cognitive biases include the memory bias, whereby wins tend to be more easily recalled than losses (Blaszczynski et al., 2008; Delfabbro et al., 2015). People who focus more closely on wins than losses can think they win more often than they do and thus consider gambling more profitable and gamble more in the future. Individuals at greater risk for experiencing gambling problems hold these beliefs to a greater extent than those without problems (Joukhador et al., 2004). This suggests that developing effective policies and practices to guide informed decision-making could positively influence gambling behaviour. However, studies indicate that the provision of information about the chances of winning, or how outcomes are determined does not change gambling behaviour (Monaghan & Blaszczynski, 2010; Newall et al., 2023a, b). More nuanced information may be required, such as personally relevant data, to achieve changes in awareness of gambling outcomes.

Consumers may be aided in countering cognitive misconceptions by having access to accurate records of their own personal wins and losses to challenge distorted views of their spending and demonstrating that they are losing more than they are winning over the long term. To make fully informed decisions, consumers need to accurately understand their prior wins and losses; that is, their net outcome resulting from previous decisions. As gambling increasingly incorporates systems to facilitate tracking of spend—including by linking spend with membership (loyalty) accounts, and cashless gambling transactions—it has become possible to provide customers with statements of their spend (i.e., activity or transaction statements; Gainsbury, 2011; Gainsbury & Blaszczynski, 2020). In some Australian jurisdictions, customers can access activity statements which provide a record of their personal wins and losses, where these are linked with accounts that accurately track gambling activity, although these typically require customers to seek these out and do not provide an overall summary. Activity statements are a form of feedback designed to assist customers in tracking their spend and are generally one of the most positively viewed harm-minimisation or responsible gambling tools (Procter et al., 2019). However, there is limited and inconsistent evidence regarding their effectiveness in increasing people’s understanding of their previous expenditure, and, as such, inform individual decision-making (Auer et al., 2023; Braverman et al., 2014; Gainsbury et al., 2025; Heirene et al., 2022). In theory, by informing about past expenditure, statements should also improve people’s ability to estimate their future expenditure. However, there has been little study of customer’s ability to prognosticate about their gambling and whether this has any connection with problem gambling status (Heirene et al., 2022; Paul et al., 2024).

Recent research has tried to understand how well people understand their recent (e.g., past month) gambling expenditure (Auer et al., 2023; Braverman et al., 2014; Heirene et al., 2022). These studies have found that many individuals cannot accurately recall their gambling outcomes and tend to substantially underestimate how much money they are losing. For example, Heirene et al. (2022) found poor rates of net outcome (i.e., overall amount won or lost) and betting frequency recall in Australian online wagerers, attributing difficulties in the former to the challenge of calculating outcomes over multiple bets. Inaccurate perceptions of past outcomes are likely to be greater for activities that involve placing many bets per session due to rapidly determined outcomes and the ability to easily re-bet any wins, such as EGMs. Evidence is mixed as to whether discrepancies between self-reported and actual outcomes are associated with problem gambling severity (Auer et al., 2023; Braverman et al., 2014; Heirene et al., 2022). Currently, the underlying cause of individual variability in recall ability remains unclear, with Heirene et al. (2022) finding no statistically meaningful predictors of (in)accuracy among demographic (age, gender) and wagering (betting frequency, net outcome) variables. Further, most research investigating the accuracy of customers’ ability to recall their gambling spend has been conducted in online settings due to the difficulty obtaining accurate objective data in land-based settings.

In the present study, we aimed to address several gaps in the literature in this area by exploring EGM customers'recall of their past gambling expenditure and predictions for future expenditure, determining the extent to which these estimations were [1] accurate in relation to actual values, [2] potential markers of risky decision making that can be used to identify individuals at-risk of gambling problems and [3] related to individual traits linked to decision-making, including impulsivity, self-control, and risk-taking propensity. Additionally, we aimed to extend the literature on recall by manipulating the conditions under which people were asked to estimate their outcome to see if this could improve accuracy. Participants were asked to estimate their spend and win values either before or after their net outcome, with all participants informed that their net outcome is the difference in these values (i.e., net outcome = win—spend). Given this, we hypothesised that those asked to estimate spend and win first would provide more accurate estimates of their net outcome. This is one of the only studies internationally to match objective EGM activity data with customers’ self-reported survey responses in a real-world gambling setting.

## Methods

We surveyed customers from a large club venue in Sydney with 720 Electronic Gaming Machines (EGMs) to collect self-report data and link these with behavioural account data obtained from the venue. The survey was hosted online using the Qualtrics platform and the venue involved had no access to the survey responses. All customers read the study information sheet and provided informed implied consent. The study materials and procedure were approved by the University of Sydney Human Research Ethics Committee (HREC Protocol No. 2023/771) and all methods were performed in accordance with relevant guidelines and regulations, including the principles outlined in the Declaration of Helsinki and the 2023 Australian National Statement on Ethical Conduct in Human Research (National Health & Medical Research Council, 2023; The World Medical Association, 2024). The methods, including recruitment strategy, participant eligibility, and outcome measures were preregistered on Open Science Framework (https://osf.io/pr35d; OSF). The hypothesis, and analysis plan relating to recall ability were also preregistered on OSF (https://osf.io/tqjcy), but we did not pre-register hypotheses or analysis plans relating to future gambling estimation given the novelty of these questions. The following hypotheses were pre-registered:

- H1: The majority of participants will fall into the “underestimated losses” group when recalling their net outcome, consistent with Heirene et al. (2022).
- H2: The difference between self-reported and actual net outcome will be statistically significant at *p* <.005 using a Wilcoxon signed-rank test.
- H3: As mentioned above, participants in the report spend-win first group will more accurately recall their past 30-day net outcome than the participants in the report spend-win last group (statistically significant difference at* p* <.005 using a Wilcoxon signed-rank test). We further specified that the spend-win first group will have a median overall percentage discrepancy between their self-reported and actual net outcome that is ≥10% less than the spend-win last group.
- H4: The median percentage discrepancy between participants’ self-reported and actual spend will be at least 110% (i.e., 10% larger than observed for net outcome recall in Heirene et al.’s (2022) study).

### Participants

Eligible customers had to have gambled at the venue at least once in the preceding 30 days, not be currently self-excluded, and have contact details available. The venue identified 4,301 customers who satisfied the inclusion criteria the day before the recruitment notice was sent. Per our pre-registration, we aimed (but were unable) to obtain a minimum of 1,000 participants, which we defined as completion up to and including all items of the Problem Gambling Severity Index (PGSI).

Of the 4,301 customers invited, 1,328 (30.90%) opened the survey. A total of 268 survey responses were recorded: 215 from the link sent via email/SMS, two from paper surveys, and 51 from the QR code link. A total of 66 responses were removed due to either ineligibility (n = 9), failure to start the survey (n = 10), overinflated self-report values greater than $599,000 (n = 2), or incorrect responses to attention check questions (n = 38). An additional 15 responses were removed as they did not include a membership number required to link responses with account data. This left 187 participants that were included in analyses, with 154 completing the past recall questions and 149 completing the future estimation questions. After linking the survey responses with account data, 43 out of the 187 responses did not have account data for the past 30-days (i.e., did not play in the venue) despite being eligible for recruitment as deemed by the venue and stating their eligibility in the survey. These 43 responses were not excluded from the analysis and actual values for net outcome, spend and win in the past 30-days were set to $0.

A recent study by Heirene et al. (in-press) used the same survey and account data involved in this study to investigate non-response bias and sample representativeness, finding that the customers who took part in the survey were mostly representative of the wider venue customer base. Comparing those who did and did not complete the survey, Heirene and colleagues found that age, gender distribution, and socio-economic status were similar between groups. Looking at six-month summaries of their account data pre-survey, those who took the survey were also comparable in their average stake/bet amount, number of betting days, and time spent on the machines to those who were invited but chose not to take part. Compared to invited non-participants, participants played less frequently between midnight and 6 AM, lost more money, had more days since their last bet, and had been a member of the venue for more years; although, all effects were small (Cohen’s *d* range = −0.22 to 0.21).

### Measures

#### Gambling Behaviour and Individual Traits

The survey included various measures assessing gambling behaviour and related-problems, individual traits, and demographic characteristics (e.g., education level, income). The measures used in analyses reported here include the Problem Gambling Severity Index (PGSI), Brief Self-Control Scale (BSCS), and impulsivity, impatience and willingness-to-take-risks measures based on the Socio-Economic Panel survey (Dohmen et al., 2011; Ferris et al., 2001; Tangney et al., 2004; Vischer et al., 2013).

The PGSI is a validated 9-item measure of problem gambling severity, with all items scored using a 4-point scale (i.e., “*0*” referring to “*never*” and “*3*” referring to “*always*”). Total scores were calculated by summing the items, with total scores of 8 + used to indicate the high-risk level (Cronbach *α* = 0.899; Ferris et al., 2001). The BSCS is a validated 13-item measure of self-control, scored on a 5-point scale (i.e., “*1*” referring to “*not at all*” and “*5*” referring to “*very much*”; Cronbach* α* for sample = 0.842; Tangney et al., 2004). Some items were reverse scored, and total scores were calculated by summing all items, with higher total scores indicating greater levels of self-control. Impulsivity, impatience and willingness-to-take risks were each measured using a single item, 11-point scale (i.e., “*0*” referring to “*very impatient*” and “*10*” referring to “*very patient*”; Dohmen et al., 2011; Vischer et al., 2013).

#### Gambling-Related Expenditure

The survey asked participants about their gambling-related expenditure over the last 30 days (pre-survey) and predicted expenditure for the next 30 days (post-survey). The questions about past expenditure were modelled on those used by Heirene et al. (2022):

1. *Approximately, how much money did you spend on pokies in the last 30 days?*
2. *Approximately, how much money did you win on pokies in the last 30 days?*
3. *Approximately, what was your net result over the last 30 days? (i.e., how much you won minus how much you spent)*

Calculating your net results – here are two examples:

- *You visited once in the last 30 days and loaded $100 into a machine, played, and withdrew $20. Your net result is -$80 ($20—$ 100). You lost $80 in the last 30 days.*
- *You load $50 on 2 separate visits into a machine and withdraw $50 on the first (you broke even) and $110 on the second (you won $60). Your net result is $60 ($160—$100). You won $60 in the last 30 days.*

The example net outcome calculation was based on Heirene et al.’s (2022) study but was adjusted to use terms and phrasing specific to EGM players. The order in which the past-30-days questions were presented differed, with participants randomised into one of two groups at a 1:1 ratio:

- Report spend-win first group: Received the questions in the following order: spend amount, win amount, [new page] net outcome
- Report spend-win last group: Received the questions in the following order: net outcome, [new page] spend amount, win amount

Questions about future gambling (next 30 days) mirrored those used for past gambling but participants were asked to estimate their expenditure for the next 30 days (e.g., *Approximately, how much money do you think will you spend on pokies in the next 30 days?).*

Participants’ responses to all questions were compared to their actual values (e.g., spend, win and net outcome) using account data provided by the venue. Participants were aware that their account data would be obtained from the venue and linked to their survey data, but they were unaware of any planned hypothesis and analyses. The venue provided account data covering six months before the first invitation for all customers invited to the survey. The data came in session format, including details for turnover (defined as total spend per session), total win, net outcome, number of games played, and time spent per session (defined by time in and time out of membership cards).

### Design and Procedure

Recruitment was conducted from December 2023 to January 2024 and involved four survey invitations sent via email and SMS by the venue. Posters and flyers with QR codes linked to the survey and paper versions of the survey were also available within the venue gaming floor. Participants, who either opened the link in the email/SMS or scanned the QR code, will be redirected to the online survey hosted in Qualtrics. Participants read the participant information sheet prior to starting the survey and provided implied consent.

The survey responses were linked to the account data using the participants’ membership numbers. For those recruited via email/SMS invitations, the survey link was unique to their membership number. Participants recruited through QR code links or completed paper surveys were asked to manually input their membership number.

Our overall project preregistration contains a detailed description of the sample identification process, recruitment strategies, recruitment notices, the survey, and the account data collected (https://osf.io/pr35d). The preregistration specific to this study contains a detailed description of the study design and analysis plan (https://osf.io/tqjcy).

### Data Analysis

Data pre-processing, variable generation, and statistical analysis were undertaken using the statistical software program Stata (version 18.0 SE; StataCorp, 2023). We generated aggregate variables summarising customers’ past and next 30-day behaviour around the day they completed the survey (e.g., past-30-day net outcome, next 30-day win, past 30-day spend/turnover). Further analysis and data visualisation was undertaken using the statistical programming language R (version 4.4.0; R Core Team, 2024). When relevant, all tests were two-tailed. Analyses scripts are stored on OSF (https://osf.io/bzutv/).

#### Variable Generation

To avoid participant burden and minimise attrition, the net outcome question had an option for participants to state *“I don’t know”.* For individuals who did not provide an estimated past or future net outcome, we calculated and imputed a value using their self-reported win and spend (imputed self-reported net outcome = self-reported win – self-reported spend^1^). For net outcome, win, and spend, we computed “absolute discrepancy” scores representing the difference between self-reported and actual values (absolute discrepancy = actual value – self-reported value), along with “percentage discrepancy” scores representing the absolute discrepancy as a percentage of the participant’s actual value (percentage discrepancy = [absolute discrepancy/actual value] × 100). For participants who did not play in the past/next 30 days, the percentage discrepancy was calculated as the absolute value of the difference between the actual value and self-reported value (e.g., percentage discrepancy =|actual net outcome – self-reported net outcome|).

#### Estimation Categories

Consistent with Heirene and colleagues'study (2022), we grouped participants based on their absolute discrepancy scores into categories (e.g., underestimated losses, overestimated spending). Participants whose self-reported and actual values exactly matched were classed as “perfectly accurate”. Also, like Heirene et al. (2022) and Auer et al. (2023), we calculated the number who were accurate in their estimations to within 10% of their actual value. A 10% margin of error was selected as a reasonable range that accounts for difficulties associated with summarising outcomes (Heirene et al., 2022).

#### Statistical Testing

##### Confirmatory Testing

We used Wilcoxon-signed rank tests to test H2 (i.e., there will be statistically significant differences between self-reported and actual values) and H3 (i.e., participants in the spend-win first group will more accurately recall their past 30-day net outcome than the participants in the spend-win last group). Absolute and percentage discrepancy scores between self-reported and actual values (i.e., net outcome, win, and spend) were non-normally distributed (Shapiro–Wilk’s test p-values < 0.05) and so were summarised using medians with ranges. As per our pre-registration, we set alpha at 0.005 for tests of our hypothesis outlined in this section to minimise type-I errors.

Spearman's non-parametric correlation tests were used to assess the association between the percentage discrepancy scores for recall and future estimation (e.g., the association between percentage discrepancy for recall of net outcome and estimation of future net outcome). An independent sample t-test was conducted to compare the percentage discrepancy between the two randomised groups (H3). The first group, referred to as Group A, was asked the total spend and win questions first and the second group, referred to Group B, was asked the total spend and win questions last. In contrast to the preceding analyses, only participants who self-reported their past net outcome (n = 106) were included in this analysis (i.e., those who said they did not know were excluded). Using only self-reported net outcome values was more appropriate when testing this H3, given the centrality of people’s own estimations of their outcome.

##### Exploratory Analyses

For exploratory, non-preregistered statistical tests alpha was set at the standard alpha level of 0.05 to maximise discoverability of relevant effects. We used six multiple linear regression models to identify the predictors of inaccurate estimations—three for recall values and three for future estimation values. The outcome in all six models was the percentage discrepancy score for the relevant value (e.g., win, spend). Tests of the statistical assumptions underlying each model were performed; outcomes from these tests and adjustments made as a result are described below.

##### Model Diagnostics – Predictors of Recall Accuracy

The residuals from the linear regression models predicting percentage discrepancy scores for spend, win, and net outcome were nonnormally distributed (Shapiro–Wilk’s test *p*-values < 0.05) and exhibited heteroscedasticity (White’s test *p*-values < 0.05). As such, a log transformation was applied to the percentage discrepancy scores. The appropriateness of this transformation was determined using a Box-Cox test (*p* > 0.05, lambda = 0). Multicollinearity was not problematic for any of the models (variable inflation factors < 5), although there were multiple influential data points (Cook’s distance > 4/*N*). All influential points were removed. Following these steps, model residuals for net outcome were normally distributed (Shapiro–Wilk’s *p* = 0.534) with no evidence of heteroscedasticity (White’s test *p* = 0.706). The residuals from the linear regression models for win and spend were still nonnormally distributed (Shapiro–Wilk’s *p*-values < 0.05). As such, non-parametric, robust linear regression models were used for these variables.

##### Model Diagnostics – Predictors of Future Estimation Accuracy

The residuals from the linear regression model predicting percentage discrepancy scores for spend, win, and net outcome were nonnormally distributed (Shapiro–Wilk’s test *p*-values < 0.05) and exhibited no evidence of heteroscedasticity (White’s test *p*-values > 0.05). The appropriateness of this transformation was determined using a Box-Cox test *(p* > 0.05, lambda = 0). Again, multicollinearity was not problematic for the models (variable inflation factors < 5), but multiple influential data points (Cook’s distance > 4/*N*) were identified and removed. The same steps were taken above to transform future estimation variables and remove influential data points; however, the residuals from the linear regression models for net outcome, win and spend were still nonnormally distributed (Shapiro–Wilk’s test *p*-values < 0.05). As such, non-parametric, robust linear regression analyses were used for these variables.

## Results

### Accuracy of Past Recall

#### Net Outcome

Table 1 outlines the distribution of participants by estimation category for past and future net outcome and Table 2 presents the characteristics of participants in each category.^2^ Of the 154 participants who reported their past-30-day net outcome, only 6 (3.9%) were perfectly accurate, noting that 3 (50%) of these were participants who self-reported a net outcome of $0 and had not played in the past-30-days. There was no significant association between accuracy categories and gender (*χ*^*2*^*(*8) = 13.51, *p* = 0.096). While a marginally non-significant difference in age across accuracy categories was observed (Kruskal–Wallis test, χ^2^(4) = 8.38, p = 0.079).

**Table 1:** Distribution of participants per type of estimation error (past recall, *N* = 154; future estimation, *N* = 149)

	Past Recall			Future Estimation		
	Net Outcome *N* = *154*	Win *N* = *153*	Spend *N* = *153*	Net Outcome *N* = *149*	Win *N* = *149*	Spend *N* = *149*
Within 10% of actual	9 (5.8%)	16 (10.5%)	4 (2.6%)	26 (17.4%)	49 (32.9%)	19 (12.8%)
Estimation Accuracy						
Perfectly Accurate	6 (3.9%)	15 (9.8%)	1 (0.7%)	26 (17.4%)	48 (32.2%)	18 (12.1%)
Underestimated Spend	-	-	101 (66.0%)	-	-	57 (38.3%)
Overestimated Spend	-	-	51 (33.3%)	-	-	74 (49.7%)
Underestimated Losses	69 (44.8%)	-	-	54 (36.2%)	-	-
Overestimated Losses	47 (30.5%)	-	-	45 (30.2%)	-	-
Underestimated Wins	18 (11.7%)	109 (71.2%)	-	5 (3.4%)	55 (36.9%)	-
Overestimated Wins	14 (9.1%)	29 (19.0%)	-	19 (12.8%)	46 (30.9%)	-

**Table 2:** Self-reported net outcome accuracy: Sample characteristics overall and by estimation type (past recall, *N* = 154; future estimation, *N* = 149)

			Favorable Bias		Unfavorable Bias	
	Overall	Accurate	Underestimated Losses	Overestimated Winnings	Underestimated Winnings	Overestimated Losses
**Past Recall**	*N* = *154*	*N* = *6*	*N* = *69*	*N* = *14*	*N* = *18*	*N* = *47*
Age	51.2 (13.6)	57.0 (9.8)	53.7 (13.4)	46.4 (14.9)	52.4 (11.6)	47.6 (13.8)
Gender						
Male	65 (42.2%)	2 (33.3%)	27 (39.1%)	7 (50.0%)	8 (44.4%)	21 (44.7%)
Female	87 (56.5%)	3 (50.0%)	42 (60.9%)	7 (50.0%)	10 (55.6%)	25 (53.2%)
Unknown	2 (1.3%)	1 (16.7%)	0 (0.0%)	0 (0.0%)	0 (0.0%)	1 (2.1%)
Self-reported outcome						
M (SD)	−2369.7 (28293.1)	−332.5 (817.0)	163.5 (1860.8)	2387.5 (5205.2)	−562.5 (1004.0)	−8457.8 (50922.5)
Mdn (IQR)	−150.0 [−600.0, 100.0]	0.0 [−20.0, 0.0]	0.0 [−400.0, 300.0]	450.0 [100.0, 2670.0]	−150.0 [−500.0, 0.0]	−500.0 [−1500.0, −200.0]
Actual outcome						
M (SD)	−746.6 (1732.9)	−332.5 (817.0)	−1653.4 (2138.9)	80.0 (226.3)	637.4 (1065.8)	−244.4 (498.0)
Mdn (IQR)	−111.5 [−1112.1, 0.0]	0.0 [−20.0, 0.0]	−862.1 [−2343.7, −354.5]	0.00 [0.00, 11.4]	225.0 [74.9, 838.0]	−0.0 [−312.1, 0.0]
Absolute discrepancy						
M (SD)	1623.1 (28231.6)	0.0 (0.0)	−1816.9 (2333.2)	−2307.5 (5215.8)	1199.9 (1906.4)	8213.4 (50662.1)
Mdn (IQR)	−60.6 [−1130.0, 350.0]	-	−1042.1 [−2387.9, −269.9]	−450.0 [−2520.5, −70.8]	511.5 [91.7, 1513.3]	400.0 [111.4, 1000.0]
Percentage Discrepancy						
M (SD)	756.0 (2380.5)	0.0 (0.0)	327.5 (1122.4)	2319.0 (5192.4)	339.8 (417.4)	1175.3 (2821.7)
Mdn (IQR)	136.1 [72.3, 400]	-	100.0 [67.2, 161.9]	734.0 [150.0, 1686.0]	145.1 [100.0, 500.5]	300.0 [100.0, 900.9]
**Future Estimate**	*N* = *149*	*N* = *26*	*N* = *54*	*N* = *19*	*N* = *5*	*N* = *45*
Age	50.84 (13.7)	46.7 (9.7)	52.4 (13.3)	50.8 (17.8)	44.0 (15.3)	52.0 (13.8)
Gender						
Male	63 (42.3%)	9 (34.6%)	27 (50.0%)	12 (63.2%)	3 (60.0%)	12 (26.7%)
Female	84 (56.4%)	16 (61.5%)	27 (50.0%)	6 (31.6%)	2 (40.0%)	33 (73.3%)
Unknown	2 (1.3%)	1 (3.8%)	0 (0.0%)	1 (5.3%)	0 (0.0%)	0 (0.0%)
Self-reported outcome						
M (SD)	233.0 (2719.5)	0.0 (0.0)	94.3 (1493.4)	2937.4 (6571.6)	−9.2 (12.28)	−580.7 (790.7)
Mdn (IQR)	0.0 [−290.6, 0.0]	-	0.0 [−100.0, 100.0]	500 [100.0, 2000.0]	−1.0 [−20.0, 0.0]	−300.0 [−500.0, −100.0]
Actual outcome						
M (SD)	−497.5 (1868.8)	0.0 (0.0)	−1378.6 (2917.1)	0.0 (0.0)	109.8 (65.4)	−5.3 (29.6)
Mdn (IQR)	0.0 [−230.0, 0.0]	-	−405.9 [−1233.7, −200.0]	0.0 [0.0, 0.0]	95.3 [79.9, 109.3]	0.00 [0.0, 0.0]
Absolute discrepancy						
M (SD)	−730.6 (3307.9)	0.0 (0.0)	−1472.8 (3358.8)	−2937.4 (6571.6)	119.0 (62.6)	575.4 (792.7)
Mdn (IQR)	0.0 [−500.0, 100.0]	-	−516.7 [−1158.4, −229.5]	−500 (−2000.0, −100.0]	109.3 [99.9, 120.3]	300.0 [100.0, 500.0]
Percentage Discrepancy						
M (SD)	611.3 (2515.0)	0.0 (0.0)	160.9 (348.2)	2937.4 (6571.6)	110.7 (13.7)	578.3 (792.0)
Mdn (IQR)	100.0 [50.0, 300.0]	-	100.0 [71.4, 124.3]	500 [100.0, 2000.0]	102.2 [100.0, 125.0]	300.0 [100.0, 500.0]

Statistics presented: Age = *M (SD);* Gender = *N (%).* All percentage discrepancy scores were converted to positive values for ease of interpretation and comparison. Abbreviations: M = mean; SD = Standard deviation; Mdn = Median; IQR = Interquartile range

The number of customers who accurately recalled their outcome increased to 9 (5.8%) when a 10% margin of error based on the participants’ actual net outcome was implemented. Partially consistent with H1, the most common type (albeit not representing a majority of participants) of estimation error regarding net outcome was underestimating losses, followed by overestimating losses and overestimating wins (see Tables 1 and 2). There were differences in the extent of inaccuracy between estimation categories, with participants who overestimated their wins (mean percentage discrepancy = 2,319%) being most inaccurate, followed by those who overestimated their losses (1,175%), then those who underestimated their wins (340%), and finally those who underestimated losses (328%).

**Fig. 1:**
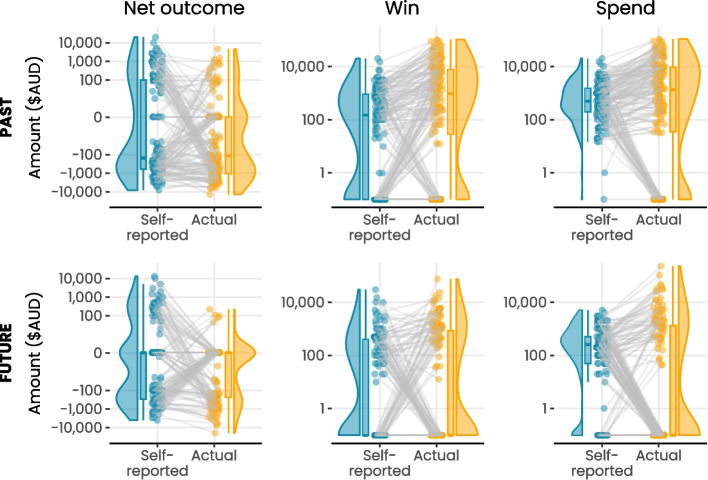
*Raincloud plots comparing self-reported and actual values for net outcome, win and spend categorised by past recall and future estimation.* These raincloud plots present a density curve, box plot, and the raw data points for self-reported and actual values, with lines connecting each person’s two values. As the natural scale for these outcomes is very large, the y-axis for win and spend is presented on a log-10 scale and the y-axis for net outcome is presented on an asinh transformed scale for better visualisation of the difference between groups

**Fig. 2:**
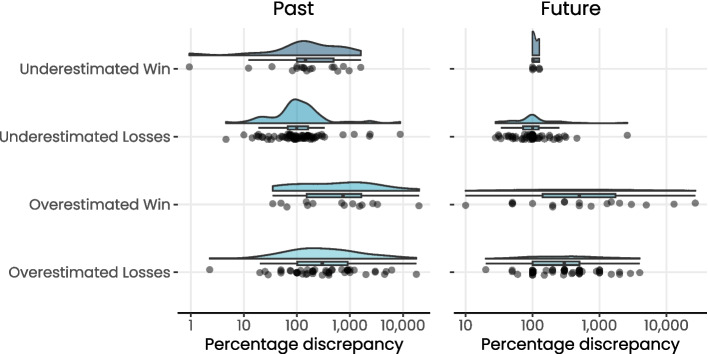
Raincloud plots of percentage discrepancy values across types of estimation error in both past recall and future estimated net outcomes. These raincloud plots present a density curve, box plot, and the raw datapoints for the absolute percentage discrepancy across the types of estimation error in both past recall and future estimated net outcomes. All values are presented on a log-10 scale to enable clear visualisation

The distribution of participants’ self-reported and actual values are visualized in Fig. 1. The overall median absolute difference between self-reported and actual net outcome was $−60.6 (*range:* −20,000.0–348,050.0) and the median percentage difference was 136.1 (*range:* 0.0–20,000.0). Contrary to H2, the difference between reported and actual values was not statistically significant at our reduced alpha level of 0.005 (*W* = 7292, *p* = 0.016). The percentage difference scores for each estimation category are visualized in Fig. 2.

#### Total Win

Of the 153 participants who reported their past 30-day win, only 15 (9.8%) were perfectly accurate, noting that these were participants who self-reported $0 win and obtained $0 actual win the past-30-days (i.e., 100% did not gamble in this window). This number increased by just one person when allowing for a 10% margin of error. The most common type of estimation error regarding wins was underestimating wins (see Table 1 and Fig. 1). On average, participants who underestimated their win did so by 86% and those who overestimated it did so by 1463%. The overall median absolute difference between self-reported and actual win was $632.65 (*range:* −20,000.0–94,270.4) and the median percentage difference was 95.1 (*range:* 0.0–20,000.0). The difference between reported and actual values was statistically significant (*W* = 1812.0, *p* < 0.001).

#### Total Spend

Of the 153 participants who reported their past 30-days spend, only 1 (0.7%) was perfectly accurate, noting that this participant self-reported $0 spend and did not play in the past 30-days. This number increased to 4 (2.6%) when a 10% margin of error was implemented. Participants most commonly underestimated their spend (see Table 1 and Fig. 1). On average, participants who underestimated their spend did so by 78% and those who overestimated it did so by 768%. The overall median absolute difference between self-reported and actual spend was $649.0 (*range:* −12,622.8–96,404.8) and the median percentage difference was 90.2 (*range:* 0.0–12,000.0). This does not support H4 (i.e., the median percentage difference value for spend would be ≥ 110%). The difference between reported and actual values was statistically significant (*W* = 2495.5, *p* < 0.001).

### Accuracy of Future Estimation

#### Net Outcome

Of the 149 participants who estimated their next-30 days net outcome, 26 (17.4%) were perfectly accurate, noting that these were all individuals who self-reported a net outcome of $0 and did not play in the next 30 days. This number did not change when a 10% margin of error was implemented. The most common type of estimation error regarding net outcome was underestimating losses followed by overestimating losses, and overestimating wins (see Tables 1 and 2). On average, participants who overestimated their win did so by 2937% and those who underestimated it did so by 111%. Participants who overestimated their losses did so by 578% and those who underestimated losses did so by 161% (see Table 2). The overall median absolute difference between self-reported and actual future net outcome was $0.0 (*range:* −27,000.0–4000.0) and the median percentage difference was 100.0 (*range:* 0.0–27,000.0). The difference between reported and actual values was statistically significant (*W* = 6876, *p* = 0.005; see Figs. 1 and 2).

#### Total Win

Of the 149 participants who estimated their next 30-day win, 48 (32.2%) were perfectly accurate, noting again that these were all participants who self-reported a win of $0 and did not play in the next30 days. This number increased to 49 (32.9%) when a 10% margin of error was implemented. Participants most commonly underestimated their wins (see Table 1 and Fig. 1). On average, participants who underestimated their wins did so by 85% and those who overestimated it did so by 1732%. The overall median absolute difference between self-reported and actual future win was $0.0 (*range:* −30,000.0–74,911.0) and the median percentage difference was 93.8 (*range:* 0.0–30,000.0). The difference between reported and actual values was not statistically significant (*W* = 4134, *p* = 0.095).

#### Total Spend

Of the 149 participants who estimated their next 30-day spend, 18 (12.1%) were perfectly accurate, noting again that these were participants who self-reported $0 spend and did not play in the next 30 days. This number increased to 19 (12.8%) when a 10% margin of error was implemented. The most common type of estimation error regarding total spend was overestimating spend (see Table 1 and Fig. 1). On average, participants who underestimated their spend did so by 84% and those who overestimated it did so by 723%. The overall median absolute difference between self-reported and actual future spend was $0.0 (*range:* −5,000.0–229,483.2) and the median percentage difference was 99.1 (*range:* 0.0–5,000.0). The difference between reported and actual values was not statistically significant (*W* = 5118, *p* = 0.468).

### Prediction of Inaccuracy of Self-Reported Gambling

#### Predictors of Accuracy in Past Recall

Table 3 presents the outcomes from three regression analyses used to identify predictors of percentage discrepancy between self-reported and actual values for past-30-day gambling. For net outcome, the number of games played was a statistically significant, negative predictor of the percentage discrepancy variable (*B* = −5.66 × 10^–5^, *t* = −2.95; *p* = 0.004), indicating that playing more games was associated with more accurate estimates. Impulsivity score was a significant, positive predictor of the percentage discrepancy variable (*B* = 0.13, *t* = 2.69; *p* = 0.008), suggesting that higher levels of impulsivity were associated with poorer recall of net outcome. For spend, higher scores on willingness-to-take risks were a significant, positive predictor of the percentage discrepancy variable (*B* = 0.07; *t* = 2.18; *p* = 0.031), indicating a greater willingness to take risks was associated with poorer recall of spend.

**Table 3:** Prediction of inaccuracy when estimating past net outcome, win and spend: Regression analyses outcomes

		*B* coefficients					Model fit
Model and terms	*B*	SE	CI^(LB)^	CI^(UB)^	Statistic	*p*-value	Adjusted *R*^*2*^
Past Recall							
Net Outcome^a^ (*n* = 122)							
PGSI score	0.03	0.03	−0.03	0.09	0.91	0.364	0.16
BSCS score	0.03	0.03	−0.03	0.09	1.11	0.268	
Impatience	−0.06	0.05	−0.16	0.04	−1.14	0.256	
Impulsivity	0.13	0.05	0.04	0.23	2.69	0.008 ***	
Willingness to take risks	0.01	0.06	−0.09	0.12	0.26	0.794	
Number of games played	−0.00	0.00	−0.00	−0.00	−2.95	0.004 ***	
Win^b^ (*n* = 123)							
PGSI score	0.00	0.02	−0.03	0.04	0.28	0.777	0.03
BSCS score	−0.01	0.02	−0.05	0.04	−0.37	0.710	
Impatience^	0.04	0.03	−0.01	0.10	1.55	0.124	
Impulsivity^	−0.05	0.04	−0.12	0.02	−1.48	0.143	
Willingness to take risks	0.03	0.03	−0.03	0.10	0.99	0.323	
Number of games played	0.00	0.00	0.00	0.00	0.74	0.462	
Spend^b^ (*n* = 124)							
PGSI score	0.02	0.02	−0.02	0.07	1.02	0.309	0.07
BSCS score	0.01	0.02	−0.02	0.04	0.53	0.599	
Impatience^c^	−0.02	0.03	−0.07	0.03	−0.71	0.480	
Impulsivity^c^	−0.01	0.03	−0.07	0.06	−0.22	0.827	
Willingness to take risks	0.07	0.03	0.01	0.13	2.18	0.031 ***	
Number of games played	−0.00	0.00	0.00	0.00	−1.66	0.100	

Predictor analysis for net outcome was done using Linear Regression (^a^). Predictor analyses for win and spend were done using non-parametric Robust Regression (^b^). All percentage discrepancy scores were converted to positive values for ease of interpretation and comparison. Abbreviations: *B* = coefficient; SE = Standard error; CI^(LB) =^ Lower bounds of 95% Confidence interval; CI^(UB) =^ Upper bounds of 95% Confidence interval; ^c^ items were reversed scored (i.e., higher scores indicated greater levels of impulsivity and impatience); *** *p* < 0.05

#### Predictors of Accuracy in Future Estimation

Table 4 presents the outcomes from three robust linear regression analyses used to identify predictors of percentage discrepancy between self-reported and actual values for next-30-day gambling. For total win, PGSI score was a significant, negative predictor (*B* = −0.10; *t* = −2.05; *p* = 0.042) indicating that higher problem gambling severity level was associated with more accurate estimates of future wins. Number of games played for the next 30 days was a significant positive predictor (*B* = 1.50 × 10^–4^*, t* = 3.52; *p* = 0.001), suggesting that playing more games was associated with more inaccurate estimates of future wins.

**Table 4:** Prediction of inaccuracy when estimating future net outcome, win and spend: Regression analyses outcomes

		*B* coefficients					Model fit
Model and terms	*B*	SE	CI^(LB)^	CI^(UB)^	Statistic	*p*-value	Adjusted *R*^*2*^
Future Estimation							
Net Outcome^a^ (*n* = 123)							
PGSI score	0.05	0.04	−0.03	0.13	1.22	0.225	0.08
BSCS score	0.07	0.04	−0.01	0.14	1.74	0.085	
Impatience^	0.45	0.07	−0.09	0.18	0.66	0.512	
Impulsivity^	0.01	0.06	−0.10	0.13	0.18	0.857	
Willingness to take risks	0.12	0.07	−0.02	0.27	1.66	0.100	
Number of games played	−0.00	0.00	−0.00	0.00	−1.53	0.128	
Win^a^ (*n* = 130)							
PGSI score	−0.10	0.05	−0.19	0.00	−2.05	0.042 ***	0.12
BSCS score	0.08	0.06	−0.04	0.19	1.31	0.191	
Impatience^	0.04	0.10	−0.16	0.23	0.37	0.711	
Impulsivity^	−0.12	0.09	−0.29	0.06	−1.30	0.197	
Willingness to take risks	−0.02	0.10	−0.22	0.18	−0.16	0.875	
Number of games played	0.00	0.00	0.00	0.00	3.52	0.001 ***	
Spend^a^ (*n* = 125)							
PGSI score	0.03	0.03	−0.03	0.10	1.01	0.316	0.06
BSCS score	0.06	0.04	−0.01	0.14	1.76	0.080	
Impatience^b^	−0.00	0.06	−0.13	0.13	−0.03	0.977	
Impulsivity^b^	−0.00	0.05	−0.09	0.09	−0.03	0.973	
Willingness to take risks	0.03	0.05	−0.08	0.14	0.50	0.621	
Number of games played	−0.00	0.00	0.00	0.00	−1.57	0.120	

Predictor analyses for net outcome, win and spend were done using Robust Linear Regression (^a^). All percentage discrepancy scores were converted to positive values for ease of interpretation and comparison. Abbreviations: *B* = coefficient; SE = Standard error; CI^(LB) =^ Lower bounds of 95% Confidence interval; CI^(UB) =^ Upper bounds of 95% Confidence interval; ^b^ items were reversed scored (i.e., higher scores indicated greater levels of impulsivity and impatience), *** *p* < 0.05

### Correlation Between Accuracy of Past Recall and Future Estimation

Table 5 presents the outcome of three Spearman’s rank correlation analyses. There was a weak, non-significant, and positive correlation between participants’ estimates of their past and future net outcome (*r* = 0.11; *p* = 0.183) and spend (*r* = 0.06; *p* = 0.492). There was a weak, non-significant, and negative correlation for past and future estimates of total win (*r* = −0.06; *p* = 0.526).

**Table 5:** Correlation of future estimates and past recall (*N* = 149)

Model and terms	*n*		Statistic	*p*-value
Net Outcome	149		0.110	0.183
Win	149		−0.052	0.526
Spend	149		0.057	0.492

Spearman’s rank correlation analysis. Abbreviations: *n* = sample size

### Comparison of Recall Accuracy across the Randomised Groups

As per H3, we compared participants who received the spend and win questions first and those who received them after the net-outcome question (these values refer to recall [past 30-days] only and not future estimation; see Table 6). Contrary to H3, the overall median discrepancy between self-reported and actual values in both groups was not statistically significant (*n* = 106, z = 0.11, *p* = 0.913). Also, contravening H3, the median percentage difference values in both groups were almost identical.

**Table 6:** Comparison of recall accuracy across randomised groups (*N* = 163)

	*Overall*	*Spend-Win First*	*Spend-Win Last*
	*N* = *163*	*N* = *82*	*N* = *81*
Absolute discrepancy			
M (SD)	2560.1 (33993.2)	−519.6 (2273.4)	6426.0 (51026.4)
Mdn (IQR)	−69.5 [−1068.2, 200.0]	−114.15 [−1130.0, 200.0]	0.0 [−974.3, 237.9]
Percentage Discrepancy			
M (SD)	925.6 (2824.2)	549.3 (1080.9)	1398.1 (4040.2)
Mdn (IQR)	129.3 [65.1, 400.0]	128.9 [72.3, 450.1]	129.8 [50.0, 300.0]

Statistics presented: Age = *M (SD);* Gender = *N (%).* All percentage discrepancy scores were converted to positive values for ease of interpretation and comparison. Abbreviations: M = mean; SD = Standard deviation; Mdn = Median; IQR = Interquartile range

## Discussion

The aims of this study were to explore people’s ability to recall past gambling expenditure and estimate their future gambling expenditure in a sample of regular EGM customers and determine the extent to which these abilities are markers of risky decision making. A unique method was used which combined self-report survey responses with behavioural account data collected by a gambling venue, enabling novel insights into the behaviours of participants and how these relate to survey responses. This is one of the first studies that the authors are aware of which have used this methodology to investigate venue-based EGM gambling.

In relation to past recall of net outcome, we found most participants underestimated actual losses. This may be attributed to the common tendency to underestimate total spend. Of those who underestimated their losses, 96% underestimated their spend (see Online Resource 2 for cross-tabulation of net outcome and spend recall estimation category frequencies). This finding is consistent with a cognitive approach to the understanding of problematic gambling which suggests that people tend to favourably bias their recall through underestimating their losses or wins being more salient (Blaszczynski & Nower, 2002; Clark, 2010). Also aligned with a model of cognitive distortions, around 20% of participants overestimated how much they were winning, which drives ongoing gambling behaviour. However, most customers won substantially more than they reported. One explanation for this is that customers are re-gambling their wins without considering this as ‘spend’ or ‘wins’. This is consistent with the ‘house money’ effect whereby customers do not consider wins their ‘own’ money until they cash out (Thaler & Johnson, 1990). This effect is potentially encouraged by the design of EGMs which automatically convert all but very large wins to credits which are held on the device rather than being paid out or separated from credit already on machines.

In relation to future gambling, participants most commonly underestimated or overestimated actual losses when predicting their net outcome. Almost 50% overestimated their spend, inconsistent with findings relating to recall of past spend wherein underestimation was most common. Similarly, many participants experienced greater wins than they predicted. However, a sizeable minority underestimated their future spend, indicating that for some gambling is not planned or intentional. Overall, participants’ estimations about past and future gambling spend were largely inaccurate. Accurate recall was almost entirely limited to those who had not gambled or did not plan to gamble in the period studied. That is, customers were only able to be accurate about their EGM gambling expenditure when they did not gamble with EGMs.

Participants with higher levels of impulsivity were more inaccurate in estimating past net outcomes and greater risk-taking was associated with greater inaccuracy in estimating past spend. These findings are consistent with the established relationship between these variables and problematic gambling – which was previously categorized as an impulse control disorder (American Psychiatric Association, 2013; Brevers & Noël, 2013; Ioannidis et al., 2019). This could be attributed to motivations of impulsive and risk-taking individuals for more immediate rewards rather than by the potential long-term consequences of their actions (Ioannidis et al., 2019; Nower & Blaszczynski, 2006). The results support the Pathways Model and other theoretical models of problematic gambling which include a biological component suggesting that there are genetically influenced traits that make individuals more vulnerable to developing and experiencing gambling harms (Blaszczynski & Nower, 2002). Individuals with greater problem gambling severity scores were more likely to be more accurate in their estimation of future wins, but not net outcome or spend or any recalled value. This effect just reached statistical significance (*p* = 0.042) and has no obvious explanation. As such, we recommend interpreting this finding with caution and attempting to replicate the effect in future research. For past recall, participants who played more games better recalled their net outcome. Repeated EGM play may lead to a greater familiarity with patterns of spending and outcomes, enabling individuals to make more accurate estimates. Further study with large samples is needed to investigate differences within a sample of regular EGM customers and compare regular with infrequent EGM customers with consideration of cognitive biases.

We used an additional manipulation in the survey to determine whether the arrangement of questions (i.e., asking about net outcome before or after total win and spend) would affect accuracy, but no significant differences in mean absolute percentage discrepancy were seen between groups. We hypothesised that those who were asked to report their spend and win first would more accurately recall their past 30-day net outcome given that the net outcome is the difference between these two variables, and this was highlighted to participants. Our findings demonstrate that even when customers are directed to think about their gambling expenditure in more granular or systematic way, this does not enhance understanding of their overall outcomes. It is possible that as participants were engaged in a research study rather than tracking outcomes for their own interest, they put minimal effort into their reporting. Further research is suggested to investigate ways to encourage consumers to think about and improve their understanding of their expenditure.

A key limitation of the current study is the relatively small survey sample size, which may have limited the statistical power required to identify significant relationships between participants’ estimations and their behaviours (number of games played), risk levels (PGSI), and personal characteristics (e.g., impulsivity, self-control). Further research with larger sample sizes is needed to investigate correlates of self-report accuracy. A recent study including this dataset by Heirene et al. (in press) revealed that the customers who took part in the survey were representative of the wider venue customer base (i.e., non-responders). However, although data were collected from a large venue, the results should not be taken as broadly representative of all EGM customers. Overall, additional research is needed to extend the results in other venues and venue types and with larger sample sizes.

### Practical implications

The ability to gamble in a sustainable manner without experiencing negative consequences is predicated on being able to gamble in line with one’s intentions and affordable time and money. If individuals are unaware of how much they are gambling, or the outcomes of gambling sessions, they have limited ability to gamble in an intentional and informed manner. This study demonstrates a strong need for regulators and gambling operators to make clear activity statements that summarise spend, wins, and net outcomes in an easily accessible and understandable manner for gambling consumers. At present, this is hindered by the use of cash and anonymous play within most EGM venues. However, our findings provide support for establishing mandatory account-based EGM play which would facilitate personalised tracking and reporting for customers (Gainsbury, 2011). Venues that offer loyalty programs for EGM play make it possible for customers to request a spend statement; however, in practice, these are rarely accessed, and they do not provide a summary of outcomes. Therefore, efforts are needed to encourage gambling customers to utilise methods to track their gambling and use these to make informed and considered decisions about future gambling. Consideration is needed for the ethical design of EGMs to enable recreational use without impairing decision-making. Some changes should not impact entertainment value, such as ensuring credits and bets are displayed as cash amounts rather than credits, issuing receipts based on money deposited and accounting for wins, and having wins automatically deposited into a separate account such they have to be consciously deposited before being regambled. Several international jurisdictions are also examining the potential impact of mandated breaks in play to provide an opportunity to disconnect from continuous betting, cognitively and emotionally, to enable more informed decisions regarding ongoing gambling. Further research is required to investigate the potential impact of these strategies, including examination of unintended consequences.

## Conclusion

Cognitive biases are accepted as playing a central role in the development and maintenance of problematic and disordered gambling. Prevention and treatment efforts often focus on enhancing consumers’ understanding of how outcomes are determined and the likelihood of winning. EGM consumers have been shown to often overestimate their chances of winning and problematic gambling is based on people spending more than is affordable gambling. This study used a novel methodology made possible by accessing customer account data matched with survey responses to understand customers’ ability to accurately track and predict their gambling behaviours. EGM customers were only able to demonstrate accurate recall of past-month gambling if they had not gambled at all. On average, customers were highly inaccurate, demonstrating minimal understanding of their expenditure. Customers do not appear recognise all of their wins and likely do not cash these out. These findings are highly problematic as consumers cannot make an informed decision to engage with a product if there is no understanding of the costs to them. Overall, there is a need to examine and alter the structural design of EGMs to enhance customers’ ability to understand the cost of play and make more considered decisions about their spend, including spending of wins.

### Analysis Code and R Packages Used

We used the ‘tidyverse’ package library for data cleaning, preparation, manipulation, and summary (Wickham et al., 2019). The ‘raindloudplots’ package was used to generate the raincloud plots (Allen et al., 2019).

## Supplementary Information

Below is the link to the electronic supplementary material.

Supplementary file1 (PDF 112 KB)

## Data Availability

The dataset for this study, excluding commercially sensitive information, is publicly available at Open Science Framework (OSF). The shared data includes participant demographics (age, gender), categorized estimates of past and predicted gambling outcomes (wins, spend, net result), randomisation assignment, and psychological measure scores (self-control, impulsivity, risk-taking propensity). Raw gambling transaction data and Problem Gambling Severity Index (PGSI) scores have been withheld due to contractual requirements related to commercial sensitivity and privacy considerations.

## Footnotes

1 This formula represents a deviation from the preregistered net outcome formula (self-reported net outcome = self-reported stake/spend – self-reported win) as the preregistered formula is incorrect and should be considered an error in the preregistration.

2 For a breakdown of the proportion of participants classified as accurate under varying error margins (10%, 20%, 30%, 40% and 50%), please refer to Online Resource 1.
