# Supplementary material for: Electronic Gaming Machine Consumers’ Understanding of Past & Future Spending: Associations with Risk, Impulsivity, Self-Control, & Problematic Gambling
Source: J Gambl Stud. 2025 Jun 9;41(4):1493–516. doi: 10.1007/s10899-025-10405-y (PMC12657582; doi:10.1007/s10899-025-10405-y)
Supplement: Supplementary file 1 — Supplementary file1 (PDF 112 KB) [file 10899_2025_10405_MOESM1_ESM.pdf]

Supplementary Material

Article Title: Electronic gaming machine consumers' understanding of past & future spending: Associations with risk, impulsivity, self-control, & problematic gambling

Journal: Journal of Gambling Studies

Author: [BLINDED FOR REVIEW]

Corresponding author: [BLINDED FOR REVIEW]

Online Resource 1

Distribution of participants per type of estimation error (past recall, N = 154; future estimation, N = 149)

	Past Recall			Future Estimation		
	Net Outcome *N = 154*	Win *N = 153*	Spend *N = 153*	Net Outcome *N = 149*	Win *N = 149*	Spend *N = 149*
Within 10% of actual	9 (5.8%)	16 (10.5%)	4 (2.6%)	26 (17.5%)	49 (32.9%)	19 (12.8%)
Within 20% of actual	15 (9.7%)	19 (12.4%)	8 (5.2%)	26 (17.5%)	50 (33.6%)	19 (12.8%)
Within 30% of actual	21 (13.6%)	22 (14.4%)	12 (7.8%)	27 (18.1%)	51 (34.3%)	20 (13.4%)
Within 40% of actual	24 (15.6%)	26 (17.0%)	15 (9.8%)	30 (20.1%)	51 (34.3%)	22 (14.8%)
Within 50% of actual	25 (16.9%)	30 (19.61%)	20 (13.1%)	34 (22.8%)	53 (35.6%)	23 (15.4%)

Online Resource 2

Frequency of participants per accuracy category for total spend in the past 30 days categorised by accuracy category for net outcome in the past 30 days (N = 154)

	Overestimated Losses *N=47*	Overestimated Win *N=14*	Perfectly Accurate *N=6*	Underestimated Losses *N=69*	Underestimated Win *N=18*
Overestimated Spend	32 (68.1%)	11 (78.6%)	3 (50.0%)	3 (4.3%)	2 (11.8%)
Perfectly Accurate	0 (0.0%)	0 (0.0%)	1 (16.7%)	0 (0.0%)	0 (0.0%)
Underestimated Spend	15 (31.9%)	3 (21.4%)	2 (33.3%)	66 (95.7%)	15 (88.2%)
